# Validation of the pregnancy-related anxiety scale (PrAS) for pregnant women in Indonesia

**DOI:** 10.1186/s12884-025-08036-7

**Published:** 2025-09-30

**Authors:** Wiwit Kurniawati, Wai Tong Chien, Nurlela Lantu, Neni Fidya Santi, Yunita Laila Astuti, Fayna Faradiena, Arie Kusumaningrum

**Affiliations:** 1 Faculty of Nursing, Universitas Indonesia, Professor Doctor Bahder Djohan Street, Depok, West Java 16424 Indonesia; 2 The Nethersole School of Nursing, Faculty of Medicine, The Chinese University of Hong Kong, Esther Lee Building, Shatin, New Territories, Hong Kong China; 3 Nursing Department, Faculty of Medicine, Universitas Negeri Malang, Cakrawala Street No.5, Malang, East Jawa 65145 Indonesia; 4 Midwifery Department, Jakarta I Health Polytechnic of Health Ministry, Wijayakusuma Raya Street 47-48, Cilandak, DKI Jakarta , 12430 Indonesia; 5 Australian Council for Educational Research Indonesia, Hang Tuah Street 9, Kebayoran Baru, DKI Jakarta, 12120 Indonesia; 6 Nursing Department, Faculty of Medicine, Universitas Sriwijaya, Indralaya Street 36, Ogan Ilir, South Sumatra 30662, Palembang, Indonesia

**Keywords:** Confirmatory factor analysis, Fear of childbirth, Indonesia, Pregnancy, Wijma delivery expectancy questionnaire

## Abstract

**Background:**

Anxiety is become a common disorder during pregnancy. The Pregnancy-related Anxiety Scale (PrAS) is a common tool for assess pregnancy anxiety. However, not yet validated in Indonesia. This study aimed to evaluate the psychometric properties of the PrAS in Indonesia.

**Methods:**

A cross-sectional study was conducted among 92 pregnant women attending a Public Health Center in Indonesia. Content validity was assessed using the Content Validity Index (CVI) and Content Validity Ratio (CVR), evaluating item relevance and inter-rater agreement. Construct validity was examined through Confirmatory Factor Analysis (CFA) to determine the underlying factor structure of the Pregnancy-Related Anxiety Scale (PrAS).

**Results:**

The mean age of pregnant women who participated was 29.12 years (SD = 4.85) with their pregnancy age mean 28 weeks. The content validity using CVI shows that 29 of 32 items are valued 1.00, the same score produced on the CVR. Indicating only 3 items need to be reviewed or revised. Regarding the construct validity, the final model of the PrAS was 7 dimensions which initially formed 8 dimensions. Although the Chi-square (χ2) index was not a good fit (df = 370, p-value = 0.0001) this multidimensional model fits the data supported by the index of Root Mean Square Error of Approximation (RMSEA) estimate was 0.058. Followed by the Comparative Fit Index (CFI) and the Tucker-Lewis Index (TLI) were 0.978 and 0.976 respectively, meaning that the goodness of fit indices were obtained. This pilot study results in a valid instrument of the Pregnancy-Related Anxiety Scale (PrAS).

**Conclusions:**

The Indonesian version of the PrAS is a valid tool for screening and assessing Indonesian women’s pregnancy-related anxiety.

**Supplementary Information:**

The online version contains supplementary material available at 10.1186/s12884-025-08036-7.

## Background

Anxiety has become a common disorder during pregnancy [1]. The prevalence of anxiety varies across countries and cultures. In Canada, 22.9% of women reported anxiety symptoms, and 15.2% were diagnosed with an anxiety disorder [2]. Other studies have found that anxiety during pregnancy ranges from 4.6 to 40.8% [1, 3]. Pregnancy-related anxiety is more closely linked to both maternal and infant outcomes than general anxiety. It encompasses worries about fetal health, the risk of fetal loss, childbirth, newborn care, and parenting [4]. This type of anxiety can have a significant negative impact on both the mother and the fetus [5], increasing the risk of complications such as preeclampsia, obstetric issues, and bonding problems [6]. Moreover, pregnancy-related anxiety can persist into the postpartum period, contributing to postpartum depression, although the severity of this depends on the social support available to the mother [7, 8]. For the newborn, maternal anxiety is associated with lower gestational age, lower birth weight, and poor cognitive development, among other issues [9].

Early detection of anxiety during pregnancy is crucial due to its potential negative impact on women’s health. To accurately measure this condition, a validated and reliable instrument is essential [8]. In Indonesia, studies on anxiety during pregnancy indicate that 20.2% of subjects experience anxiety at varying levels of severity. Study found that 53.3% of respondents reported feeling anxious, with higher levels of anxiety among pregnant women with higher education, those in their first trimester (< 19 weeks), and working women [10]. Despite this, anxiety during pregnancy remains under-assessed in delivery services, affecting both maternal and fetal well-being. Therefore, a prompt and effective instrument, such as the Pregnancy-related Anxiety Scale (PrAS), is needed in the Indonesian context. Cultural differences in Indonesia may require adaptations to the original PrAS to ensure its relevance and validity.

The PrAS is an instrument developed by Robyn Brunton and colleagues in 2018 to measure pregnancy-related anxiety. It serves as a screening tool, where higher scores indicate greater anxiety related to pregnancy (Brunton et al., 2018) [11]. Participants respond to 11 items on a scale from 1 (“never”) to 4 (“very often”), with reverse-scored items [11]. Several other instruments are available to measure pregnancy-related anxiety, but it is essential to ensure that these tools capture the unique aspects of pregnancy-related anxiety. The PrAS was designed to address these specific concerns.

The PrAS consists of eight dimensions: Childbirth Concerns, Body Image Concerns, Attitudes towards Childbirth, Worry about Self, Acceptance of Pregnancy, Attitudes towards Medical Staff, Avoidance, and Baby Concerns. The “Childbirth Concerns” dimension includes items related to maternal fears and concerns about childbirth. The “Attitudes towards Childbirth” dimension assesses the mother’s readiness—physically, mentally, and emotionally—to face the labor process.

This adaptation study aims to evaluate the psychometric properties of the Indonesian version of the PrAS (I-PrAS). The objectives of the study are to: (a) validate how well the items cover all relevant aspects of content (content validity) using CVI and CVR; and (b) test the construct validity of the Indonesian version of the PrAS to determine whether the eight-factor structure of the original PrAS scale fits the Indonesian sample using Confirmatory Factor Analysis (CFA). The results of this study will provide important insights for the further development and validation of the Indonesian version of the PrAS.

## Methods

### Study design

A cross-sectional study was conducted to adapt the Pregnancy-Related Anxiety Scale (PrAS) among 92 pregnant women with singleton pregnancies at a public health center in Jakarta, Indonesia, in November 2023. The study received ethical approval from the Institutional Review Board of the Faculty of Nursing, Universitas Indonesia. Written informed consent was obtained from each participant after a clear explanation of the study’s objectives and procedures.

### Sample and study participants

Pregnant women with a singleton pregnancy who were receiving antenatal care at an antenatal clinic were screened and recruited by the investigators. A convenience sampling method was used for participant selection. The inclusion criteria were mothers with a singleton pregnancy who spoke and understood Bahasa. The exclusion criteria included mothers with obstetric complications (e.g., preeclampsia or hemorrhage) or those with a history of mental illness. The study was conducted at a public health center in Indonesia, selected due to the high number of antenatal visits.

A total of 92 participants were selected based on the specific inclusion criteria targeting pregnant women at 28 weeks gestation in selected clinics in Jakarta, which limited the available population. While there is no universally agreed-upon rule for sample size in Confirmatory Factor Analysis (CFA), methodological literature suggests that a minimum ratio of 5 participants per item may be acceptable under certain conditions (Mundfrom, Shaw, & Ke, 2005). The Pregnancy-Related Anxiety Scale (PrAS) consists of 32 items, which would indicate a recommended minimum sample size ranging from 160 participants or more for optimal stability. However, due to practical constraints during the data collection period, including limited time and accessibility, a sample of 92 participants was obtained. Although this sample size may be considered modest, it falls within the lower bound of acceptable ratios used in exploratory and confirmatory factor analysis under constrained research settings, and was therefore deemed sufficient for preliminary validation purposes [12, 13]. The claim that “a minimum of 2 to 5 participants per item of an instrument” is acceptable is consistent with standard methodological guidance, where sample size recommendations range from 2 to 5 respondents per item (Gorsuch, 1983; Hatcher, 1994; Pett et al., 2003; Costello & Osborne, 2005). For a 32-item instrument, this implies a required sample size ranging from 64 to 160 participants, depending on communalities and factor structure. The chosen sample size of 92 participants falls within this acceptable range and is considered adequate for conducting content and construct validity assessments, including confirmatory factor analysis (CFA) [12, 13]. Additionally, according to Lwanga and Lemeshow (1991), small sample sizes are acceptable in health studies when targeting narrowly defined populations. Similarly, Bartlett et al. (2001) and Roscoe (1975) suggest that sample sizes between 30 and 100 can be sufficient for exploratory or scale validation studies, particularly when the study population is constrained. The National Academy of Sciences (2001) also recognizes that small clinical samples are appropriate in specialized health research where larger populations are inaccessible, ensuring methodological rigor despite sample limitations.

### Procedure

#### Translation and back translation of the PrAS

Cross-cultural research instrument validation is essential due to the diversity of the global population. To ensure the quality of the instrument, the researcher arranged for translation from English into Indonesian using the following steps.

##### Selecting Well-Qualified translators

For this study, the original instrument was translated from English into Bahasa Indonesia and then back-translated into English. To ensure a high-quality translation, it is essential to use well-qualified translators [14]. The translators for this study were fully bilingual and sufficiently educated to understand the concepts of pregnancy-related anxiety. They were independent and not authors of this study. The first translator (AY) is an expert in English education and has studied anxiety. She translated the PrAS from English into Bahasa Indonesia. The second translator (AM), who performed the back-translation, is a faculty member in the Nursing Department. She has conducted studies focusing on psychological nursing, including her published papers titled “Reducing Psychological Distress in Patients Undergoing Chemotherapy” and “Fear of Cancer Recurrence and Social Support Among Indonesian Gynecological Cancer Survivors.” The second translator then translated the Indonesian version of the PrAS back into English.

##### **Translating the Instrument**

a According to Brislin (1970), the most common and highly recommended procedure for translating a questionnaire is translation and back-translation. The steps for translating an instrument include translating from the source to the target language, blindly translating back from the translated version to the source language, and comparing the two versions in the original language (15).

1. **Translating from the source to the target language.** The first translator translated the original English version into the target language, Bahasa Indonesia, which is the official language of Indonesia.
2. **Blindly translating back from the target to the source language. **Back-translation involves translating the newly translated version back into the original source language without looking at the original [15]. The second translator back-translated the Indonesian version provided by the first translator into English. The forward translator and back-translator did not discuss the translation during this stage to avoid the back-translator being influenced by the original English version of the questionnaire.
3. **Comparing the two versions in the original language.** The equivalence of the source and target language versions of the instrument must be evaluated. If the two English versions of the questionnaire are identical, the translated version (in this case, the Indonesian version) may be considered equivalent to the original [15]. Therefore, the researcher conducted a panel discussion to compare the original English version, the back-translated English version, and the final Indonesian version to ensure consistency.

##### **Measure**

a We have conducted a pre-test of the Indonesian version of the Pregnancy-related Anxiety Scale (I-PrAS) with a small group of participants to assess the clarity and understanding of the scale items. Based on the pre-test results, any unclear or confusing items were revised to ensure they were comprehensible for the target population.
b Regarding the use of Google Forms as a data collection method, we acknowledge that it may not be the most appropriate approach for this type of study, particularly given the sensitive nature of the topic. To address this, we revised our data collection procedure to include more direct supervision and guidance from the research team during the administration of the survey. Specifically, we plan to conduct the survey online via Google Forms, but with the researcher available to answer questions and ensure that participants understand the items properly, providing clarification as needed. This ensures the survey remains accessible while also providing the necessary support to participants during data collection.

**Fig. 1:**
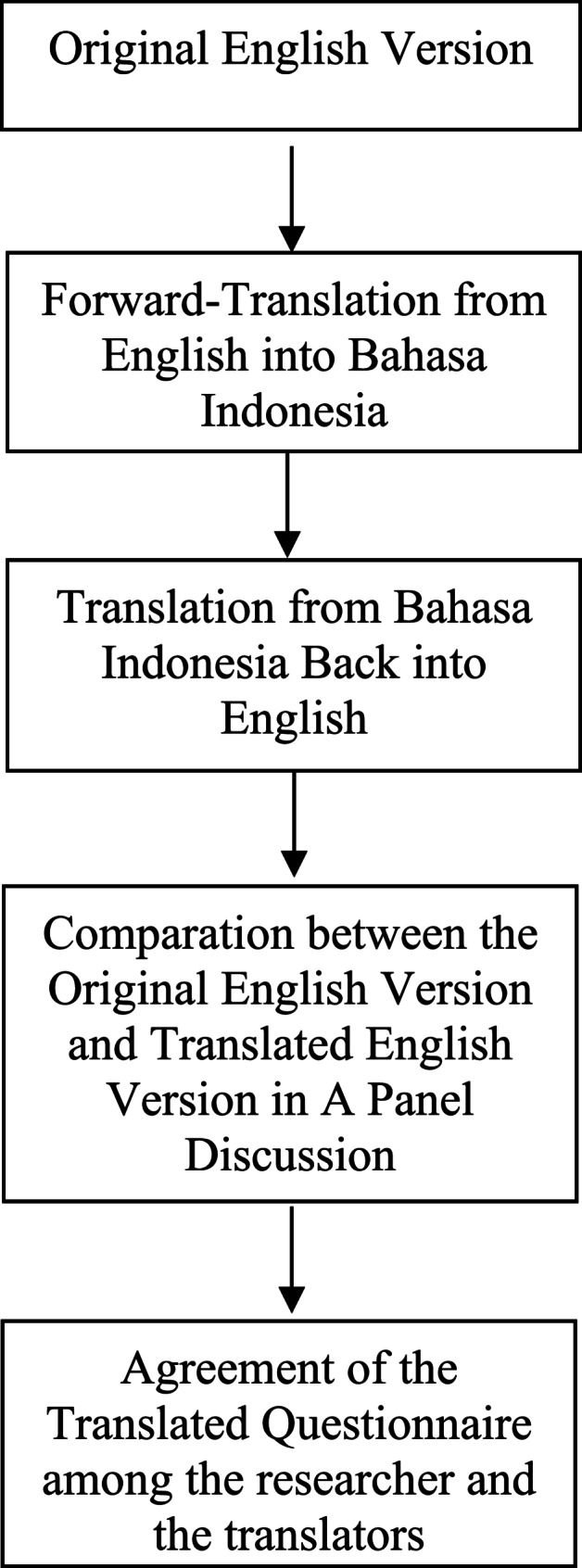
Flow Chart of Translation and Back Translation Process

The process of translation and back translation of Pregnancy-related Anxiety Scale is described in Fig. 1.

#### Instrument content validity assessment of the PrAS

To assess the content validity of the Indonesian version of the PrAS, the Content Validity Index (CVI) and Content Validity Ratio (CVR) were calculated based on evaluations by six subject-matter experts. These six experts included two maternity nurses, three lecturers in maternity nursing, and one obstetrics and gynecology doctor. According to Sousa, et al. (2011) and Polit, et al. (2007), the minimum number of experts required for such an evaluation is three [14, 16].

The experts were asked to evaluate each item in the Indonesian version of the PrAS in terms of its relevance, clarity, and cultural appropriateness. A four-point Likert scale was used for rating relevance (1 = not relevant to 4 = highly relevant), as recommended by Lynn (1986).

We calculated the Item-Level Content Validity Index (I-CVI) by determining the proportion of experts rating each item as either 3 or 4. An item was considered acceptable if its I-CVI was ≥ 0.78, based on Lynn’s criteria for a panel of six experts.

The CVI was used to determine item relevance based on expert ratings. According to standard guidelines, an Item-Level Content Validity Index (I-CVI) score of 1.00 is optimal when six experts are involved. Items with I-CVI < 0.78 are considered for revision or elimination [16].

In addition, we calculated the Content Validity Ratio (CVR) for each item using Lawshe’s (1975) formula: CVR = (Ne – N/2)/(N/2), where Ne is the number of panelists rating the item as “essential”, and N is the total number of panelists. A CVR value close to 1.00 indicates strong agreement on item essentiality. For six experts, a CVR of at least 0.99 is considered excellent [17].

In this study, of the 32 items evaluated, 29 items achieved an I-CVI and CVR score of 1.00, indicating excellent content validity. Three items (items 30, 31, and 32, related to women’s attitudes towards medical staff) had a lower I-CVI (0.67) and CVR (0.83), suggesting the need for revision before full-scale deployment. The content validity index (CVI) in this study, rated by the six experts, ranged from 0.83 to 1, with an average of 0.984. The scale’s content validity (S-CVI) average for an instrument should be at least 0.90. Additionally, according to Polit and colleagues, an item-level content validity (I-CVI) greater than 0.50 is acceptable. Specifically, for six experts, an item with an I-CVI of 0.67 is considered “fair,” while 0.86 and 1.00 are considered “excellent,” according to the kappa evaluation criteria [16]. These results indicate that the PrAS is valid and reliable for use in Indonesia.

### Measurement

A pilot test was conducted with 92 pregnant women to assess the clarity, comprehensibility, and overall adequacy of the final version of the PrAS instrument. The questionnaire was administered via Google Forms for reading instructions, reflecting on the items, and selecting responses. For participants with limited literacy, the investigator read the items aloud in Bahasa Indonesia and provided assistance in completing the form.

Psychometric testing included both content and construct validity assessments. Content validity was evaluated using the Content Validity Index (CVI) and Content Validity Ratio (CVR), while construct validity was examined using Confirmatory Factor Analysis (CFA). The 30-minute estimate reflects typical completion conditions and is intended to inform the scale’s practical application in community and clinical settings.

### The pregnancy-related anxiety scale (PrAS)

The Pregnancy-related Anxiety Scale (PrAS) was developed by Robyn Brunton and colleagues to measure anxiety related to women’s feelings during pregnancy. The instrument consists of 32 items and uses a four-point Likert scale, with responses ranging from 1 to 4: 1 = not at all, 2 = occasionally, 3 = quite often, and 4 = very often. The highest and lowest scores represent the opposite extremes of a pregnant woman’s [11]. The 32-item questionnaire is divided into eight main subscales: items 1 to 6 assess childbirth concerns; items 7 to 11 describe body image concerns during pregnancy; items 12 to 14 correspond to attitudes towards childbirth; items 15 to 20 reflect worries about self; items 21 to 23 indicate concerns about the baby; items 24 to 26 assess acceptance of pregnancy; items 27 to 29 are related to avoidance of vaginal delivery; and items 30 to 32 focus on attitudes towards medical staff (Brunton, Gosper, & Dryer, 2021). The total possible score on the PrAS is 128, with a cut-off point of 75.50 indicating a high level of anxiety in a pregnant woman. According to Brunton et al., the original PrAS demonstrated excellent internal consistency, with a full-scale α = 0.92 and subscale reliabilities ranging from 0.84 to 0.95.

#### Demographic variables

Demographic data including age, employment status, educational level (background), pregnancy age, and pregnancy sequence were assessed using a structured questionnaire.

### Statistical analysis

Data analysis was conducted using the Statistical Package for the Social Sciences (SPSS) version 26 (IBM, 2019) and MPlus version 8.0 [18]. Descriptive statistics, including frequency, mean, and standard deviation (SD), were used to summarize the participants’ characteristics. During the development phase of the Indonesian version of the Pregnancy-related Anxiety Scale (I-PrAS), each of the 32 items of the instrument was evaluated and rated by six panelists (subject matter experts). Content validity was assessed to measure the relevance of the construct for the PrAS, and construct validity was evaluated using Confirmatory Factor Analysis (CFA). CFA was performed to determine whether the Indonesian version retained the same factor structure as the original PrAS.

The weighted least squares mean and variance adjusted (WLSMV) estimator provides robust results that do not assume normally distributed variables, making it the best option for modeling categorical or ordered data [19]. Additionally, the Bayesian estimator was used as the fit index model. Bayesian approaches for estimating multilevel latent variable models are particularly beneficial in small samples [20]. Prior specification for more stable Bayesian estimation of multilevel latent variable models in small samples has been compared in two different approaches [20].

Model fit was evaluated using the following indices: Root Mean Square Error of Approximation (RMSEA), Comparative Fit Index (CFI), Tucker-Lewis Index (TLI), and Standardized Root Mean Square Residual (SRMR). Following conventional cut-off criteria, RMSEA values ≤ 0.06, CFI and TLI values ≥ 0.95, and SRMR ≤ 0.08 indicate a good model fit [21, 22].

Goodness-of-fit indices were used to confirm the model fit, namely, a Chi-square test (χ2) and its p-value (≥ 0.05 = acceptable fit; ≥0.10 = good fit), which is sensitive to large sample size. Therefore, there are in this study other indices included root mean square error of approximation (RMSEA: ≤0.08 = acceptable fit; ≤0.05 = good fit), comparative fit index (CFI: ≥0.85 = acceptable fit; ≥0.95 = good fit), and Tucker-Lewis index (TLI: ≥0.80 = acceptable fit; ≥0.95 = good fit) were considered in this study. At least three adequacy indices with values within the acceptable ranges were considered in analyzing the goodness of fit of data.

## Result

### Characteristic of participants

In total, 92 pregnant women have participated in this study. The majority of women were older ≥ 25 years (87.61%) with the mean of participants being 29.12 (SD = 4.75) and the pregnancy age 28 weeks. The participants were employed (36.96%) and had a secondary education (55.43%) (See Table 1). Most of the women experienced their first pregnancy and second pregnancy (69.57%).

**Table 1:** Descriptive statistics

Variables	Frequency	Percentage (%)
*Employment Status*		
Not Employed	57	61.96%
Employed	34	36.96%
*Education Level*		
Primary Level	2	2.17%
Secondary Level	51	55.43%
Tertiary Level	37	40.22%
*Pregnancy Sequence*		
First Pregnancy	34	36.96%
Second Pregnancy	30	32.61%
Third Pregnancy	19	20.65%
Fourth Pregnancy	8	8.70%

### Translation of the PrAS

To ensure the accuracy and cultural relevance of the translated scale, we followed Brislin’s (1970) back-translation method [15]. The process began with the forward translation of the PrAS from English into Bahasa Indonesia, followed by a blind back-translation into English. The original and back-translated versions were then compared to identify any discrepancies. Throughout this process, the researchers, translators, and the primary researcher discussed differences in terms and sentence structure to ensure both linguistic and cultural alignment.

In particular, differences were noted in items 3, 4, and 5. For item 3, the forward translator included the word “very” in the Indonesian version, which did not appear in the original English version. After discussion, the researcher decided to remove the word “very” from both the Indonesian and back-translated versions to maintain consistency with the original text.

In item 4, the back-translator used the word “stiff,” while the original English version used “restrain.” This discrepancy arose because the forward translator had translated “restrain” as “stiff” in Indonesian. To resolve this, the researcher and translators agreed to revise the Indonesian version so that it aligned better with the English term.

Finally, in item 5, the back-translator used “aggrieved” instead of the original term “harmed.” Given that “harmed” refers to being physically injured, which differs from “aggrieved,” the researcher determined that the translated version should use a term that more closely aligns with the meaning of “harmed.” After further discussion, the term was revised to better reflect the original intent.

Ultimately, after reviewing these items and resolving the discrepancies, the researcher and translators reached an agreement on all items, ensuring the accuracy and cultural appropriateness of the final translation.

Conceptual Definitions and Comparison of Sub-Dimensions.

The Indonesian version of the PrAS (I-PrAS) was adapted to ensure that the sub-dimensions align with the cultural context of Indonesia. The seven sub-dimensions of the I-PrAS were conceptually defined and revised as follows:

1. Childbirth Concerns (gb): This sub-dimension remains largely consistent with the original PrAS, focusing on maternal worries and fears about the childbirth process.
2. Body Image Concerns (bi): This sub-dimension was slightly adjusted to better reflect concerns about bodily changes specific to Indonesian cultural contexts.
3. Attitudes Towards Childbirth (ac): The sub-dimension was adapted to incorporate cultural views of childbirth, emphasizing the role of family and traditional practices in the Indonesian context.
4. Worry About Self (sf): This dimension remained conceptually similar to the original, but we ensured that it addressed both personal health concerns and social expectations related to pregnancy in Indonesia.
5. Concerns About the Baby (bb): We maintained the original definition but included more specific worries relevant to Indonesian mothers, such as concerns about the health of the baby in relation to local health practices.
6. Avoidance of Vaginal Delivery (an): This was introduced as a new sub-dimension to address societal influences on women’s choices regarding delivery methods, a concern that is prominent in Indonesian culture.
7. Attitudes Towards Medical Staff (ms): This sub-dimension was revised to reflect Indonesian views on healthcare professionals, particularly the relationship between patients and doctors in the Indonesian healthcare system.

These sub-dimensions were compared to the original scale, ensuring that cultural nuances were addressed while preserving the essence of the original measure.

### Content validity result

The Pregnancy-Related Anxiety Scale (PrAS) was evaluated for content validity through a structured assessment involving six experts: two maternity nurses, three academic lecturers in maternity nursing, and one practicing obstetrician. Each expert independently rated all 32 items in the scale for their relevance to the construct of pregnancy-related anxiety using a 4-point Likert scale (1 = not relevant, 2 = somewhat relevant, 3 = quite relevant, 4 = highly relevant).

To quantify expert agreement, two indices were calculated: the Content Validity Index (CVI) and the Content Validity Ratio (CVR). The CVI was computed at the item level (I-CVI) by dividing the number of experts rating the item as either 3 or 4 by the total number of experts. An I-CVI of 1.00 indicates perfect agreement among all experts on an item’s relevance. The CVR was calculated using the formula CVR = (Ne – N/2)/(N/2), where Ne is the number of experts who rated the item as “essential” and N is the total number of experts. According to Lawshe’s criteria, with six experts, a minimum CVR value of 0.99 is considered acceptable for strong content validity.

Based on this analysis, 29 out of the 32 items showed excellent content validity, with both I-CVI and CVR scores of 1.00, indicating complete agreement among the panel of experts. These items were deemed highly relevant and essential for assessing pregnancy-related anxiety among Indonesian women.

Conversely, three items—item 30, item 31, and item 32—demonstrated comparatively lower scores (CVI = 0.67; CVR = 0.83). These items specifically address perceptions and attitudes toward interactions with healthcare professionals during pregnancy. The reduced scores suggest that the experts had mixed opinions about the importance and clarity of these items in capturing the construct. Although these values are below the optimal threshold, they still fall within an acceptable range for preliminary validation. Therefore, these items are flagged for revision and cultural adaptation before final implementation of the scale in larger populations.

### Content validity ratio (CVR)

Each item of PrAS is counted by the formula CVR=(N_e_ - N/2)/(N/2), in which the N_e_ is the number of panelists indicating “essential” and N is the total number of panelists (in this study evaluated by 6 panelists). From 32 items, only 3 items (30, 31, 32) in the woman’s attitudes towards medical staff dimension show the value of CVR 0.83, while others are 1.00. The closer to 1.0 the CVR is, the more essential the object is measured to be. Contrariwise, the closer to −1.0 the CVR is, the more non-essential it is [23].

### Content validity index (CVI)

The content validity index (CVI) is related to the number of experts. Reflecting this study where there are 6 or fewer experts, the I-CVI must be 1.00—that is, all experts must agree that the item is content valid [16]. From 32 items, only 3 items (30, 31, 32) in the woman’s attitudes towards medical staff dimension show the value of CVI 0.67, while others are 1.00. It shows that the three items need to be revised before entering the data collection phase.

### Confirmatory factor analysis

**Fig. 2:**
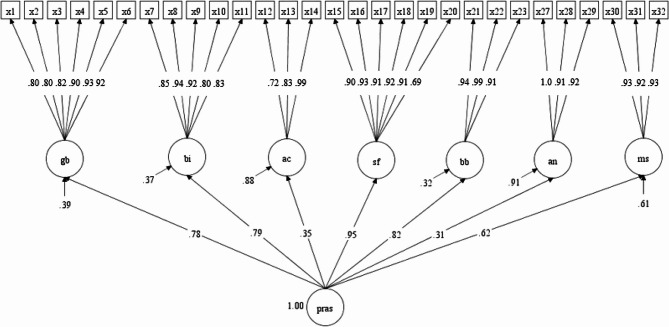
The multidimensions of PrAS model. Note: *gb* (childbirth), *bi* (pregnant woman’s body image), *ac* (attitudes towards childbirth), *sf* (worry about self), *bb* (concerns to the baby), *an* (woman’s avoidance of vaginal delivery), and *ms* (woman’s attitudes towards medical staff)

Confirmatory Factor Analysis (CFA) was conducted to test the first-order multidimensional structure of the Indonesian version of the Pregnancy-Related Anxiety Scale (PrAS), based on the original 8-factor model. The initial model showed an acceptable fit (RMSEA = 0.064). After removing one underperforming dimension (“woman’s acceptance of pregnancy”), the revised 7-factor model demonstrated a good fit to the data (RMSEA = 0.058, CFI = 0.978, TLI = 0.976). Each latent factor was supported by multiple items, and all standardized factor loadings were statistically significant (*p* < 0.001). Figure 2; Table 2 present the final model and factor loadings.

The eighth dimension, *woman’s acceptance of pregnancy*, comprising items 24–26, was removed from the model due to its poor performance in the CFA. Specifically, it yielded a negative standardized factor loading (λ = − 0.038) and failed to contribute meaningfully to the overall construct of pregnancy-related anxiety. This decision was supported by conceptual concerns, as the items were less aligned with anxiety-related domains in the Indonesian cultural context. The final model therefore retained 29 items across 7 dimensions.

The final model eliminated one of the dimensions, named woman’s acceptance of pregnancy (item 24, item 25, and item 26) with the loading of −0.038 in measuring PrAS. After the model fit was tested, the next step was to identify each dimension factor loading (λ) or coefficient which indicates the contribution of the items to the construct being measured (pregnancy-related anxiety). Initially, the 32-item measure yielded 10 items due to the eliminated dimension, resulting in 29 final items divided in 7 dimensions. Table 2 below shows the information of the standardized dimensions and its significance.

**Table 2:** Standardized model results of PrAS

Dimension	Coefficient (λ)	Standard Error (S.E)	T-Value	*P*-Value	Sig.
Childbirth (gb)	0.782	0.046	17.054	0.001	*
Pregnant woman’s body image (bi)	0.792	0.046	17.172	0.001	*
Attitudes towards childbirth (ac)	0.351	0.088	4.008	0.001	*
Worry about self (sf)	0.955	0.031	30.468	0.001	*
Concerns to the baby (bb)	0.823	0.039	20.929	0.001	*
Woman’s avoidance of vaginal delivery (an)	0.308	0.104	2.972	0.001	*
Woman’s attitudes towards medical staff (ms)	0.622	0.068	9.206	0.001	*

Note: *p*-value < 0.05 (Significant)

## Discussion

This study aimed to validate the Indonesian version of the Pregnancy-Related Anxiety Scale (PrAS) by examining its content and construct validity through expert assessment and Confirmatory Factor Analysis (CFA). The main findings demonstrate that the Indonesian PrAS version possesses strong psychometric properties. Specifically, the content validity indices (CVI and CVR) indicated high agreement among experts, with 29 of 32 items achieving perfect scores. Construct validity, assessed using CFA, supported a revised seven-factor model after excluding one underperforming dimension, resulting in a model with excellent fit indices (RMSEA = 0.058, CFI = 0.978, TLI = 0.976). All standardized factor loadings were statistically significant (*p* < 0.001), confirming the scale’s multidimensional structure in the Indonesian context.

These findings align with the original validation study by Brunton et al., which also reported high internal consistency (α = 0.92) and strong factor structure for the eight dimensions of PrAS. Similar validation efforts in other countries, such as Iran and Turkey, have confirmed the multidimensional nature of pregnancy-related anxiety and demonstrated comparable psychometric strengths. For example, the Turkish version of PrAS retained all original dimensions but required cultural adaptation for specific item wordings. In contrast, the current study found that one dimension—woman’s acceptance of pregnancy—underperformed in the Indonesian context, leading to its exclusion and improving model fit.

This finding is consistent with validation studies conducted in other countries. In China, Feng et al. (2025) conducted a cross-cultural adaptation of the PrAS and emphasized the need for refinement through translation and cognitive debriefing processes. Although not focused on factor structure alone, their work highlights the contextual influences that shape how anxiety is expressed and measured [24]. Meanwhile, Xie et al. (2022) validated the Chinese version of the PRAQ-R2, using CVI, CVR, and CFA, and found that several items required modification to better align with local sociocultural expressions [25]. Similarly, Kurt and Arslan (2021) confirmed the validity of the Turkish PrAS following cultural adaptation [26]. Dryer et al. (2022) also reinforced the multidimensionality of PrAS by validating its screener version across diverse populations [27].

Additional cross-cultural insights have emphasized that pregnancy-related anxiety manifests differently across sociocultural environments. Bright and Premji (2021) noted that the expression of anxiety during pregnancy is influenced by cultural narratives, healthcare expectations, and social support systems, which directly impacts how women interpret and respond to survey items [28]. Therefore, culturally grounded revisions to instruments like the PrAS are not only appropriate but necessary to ensure accurate measurement.

The current study contributes to this growing body of research by confirming the psychometric adequacy of a culturally adapted version of the PrAS for use in Indonesia. The results suggest that while the underlying construct of pregnancy-related anxiety is consistent across populations, certain dimensions or items may not fully capture culturally nuanced experiences unless adapted. This supports the argument that CVI, CVR, and CFA should be integrated as standard procedures in all cross-cultural scale validation efforts [12].

These comparisons suggest that while the core structure of PrAS is generally robust across cultures, certain dimensions may require modification or removal to ensure relevance and clarity within different cultural settings. The retention of seven dimensions in the final Indonesian model confirms that the PrAS is adaptable and maintains its conceptual integrity when applied to diverse populations.

### Validity test of the Indonesian version of PrAS

Factor analysis is a widely used method to test the construct validity of an instrument. In particular, Confirmatory Factor Analysis (CFA) is used to examine the extent to which observed variables reflect the expected latent constructs, ensuring that each item truly measures the intended dimension of the instrument. When a proposed model does not demonstrate acceptable fit indices, it may indicate that certain items or dimensions are not contributing appropriately to the underlying construct and should therefore be removed to avoid measurement bias (29).

In the present study, construct and content validity of the Indonesian version of the Pregnancy-related Anxiety Scale (I-PrAS) were assessed. Initially, the instrument consisted of 32 items across 8 dimensions. However, based on both content and construct validity testing, one dimension—woman’s acceptance of pregnancy—was removed, resulting in a final version containing 29 items across 7 dimensions.

The results of the Content Validity Ratio (CVR) showed that only three items from the woman’s attitudes towards medical staff dimension had a CVR value of 0.83, while the remaining items scored 1.00. Similarly, the Content Validity Index (CVI) results indicated that the same three items had a CVI value of 0.67, whereas all other items scored 1.00. These findings suggest that the three items in that dimension require further revision and refinement before proceeding to the next phase of data collection.

Construct validity was supported by the CFA results, with goodness-of-fit indices meeting acceptable thresholds: RMSEA (Root Mean Square Error of Approximation), CFI (Comparative Fit Index), and TLI (Tucker-Lewis Index) all indicated a good model fit. Thus, the revised seven-dimensional structure of the I-PrAS was considered valid for use in the Indonesian context. Nevertheless, we recommend further evaluation and cultural adaptation of the excluded dimension for potential inclusion in future applications.

### Study limitations and future directions

One limitation of this study is that it was conducted in a single region of Indonesia—specifically, Jakarta. Jakarta has unique demographic characteristics as the capital city of Indonesia, reflecting the diversity of the Indonesian population. As a place where people from various regions migrate to stay and work, this may contribute to a higher risk of anxiety among pregnant women. While the study sample from Jakarta does reflect a broad diversity of Indonesian mothers, it is limited to an urban context. Therefore, the findings may not be generalizable to other regions of Indonesia, particularly rural areas.

To further validate the I-PrAS, future studies should include a larger and more geographically diverse sample that better represents the entire country. A more extensive study would provide a broader understanding of pregnancy-related anxiety across different regions and further validate the I-PrAS for use in Indonesia.

While Confirmatory Factor Analysis (CFA) was used in the current study to confirm the factor structure, a future study may benefit from conducting Exploratory Factor Analysis (EFA) on a larger and more diverse sample to further explore the underlying dimensions and cultural adaptations of the PrAS in the Indonesian context.

This study was conducted in Jakarta, an urban area with a diverse demographic due to migration from various regions in Indonesia. While this reflects the diversity of the Indonesian population, it may limit the generalizability of the findings to rural or other regional populations. Therefore, future research should aim to include a larger, more representative sample from different regions of Indonesia to further validate the I-PrAS across the entire country.

Additionally, the woman’s acceptance of pregnancy sub-dimension, which was eliminated in this study due to low factor loading, requires further investigation. More work is needed to refine this sub-dimension and assess how it can be adapted to better fit the Indonesian cultural context. Future studies should explore how cultural factors influence this sub-dimension and consider modifications that would make it more relevant for the Indonesian population.

## Conclusion

The Indonesian version of the Pregnancy-related Anxiety Scale (I-PrAS) with 7 dimensions is a valid tool for screening and assessing pregnancy-related anxiety among Indonesian women, as demonstrated in this adaptation study. However, to ensure its broader applicability, future research should consider expanding the sample to include a more representative group of pregnant women from diverse regions of Indonesia. This would allow for the instrument’s potential use on a national scale.

## Supplementary Information

Supplementary Material 1.

## Data Availability

No datasets were generated or analysed during the current study.

## Abbreviations

CFA: Confirmatory Factor Analysis
PrAS: Pregnancy-related Anxiety Scale
CFI: The Comparative Fit Index
TLI: Tucker-Lewis Index
RMSEA: Root mean square error of approximation
WMLSV: weighted least squares mean and variance adjusted

## References

[1] Falah-Hassani K, Shiri R, Dennis C-L. The prevalence of antenatal and postnatal co-morbid anxiety and depression: a meta-analysis. Psychol Med. 2017;47(12):2041–53. 28414017 10.1017/S0033291717000617

[2] Dennis C-L, Falah-Hassani K, Shiri R. Prevalence of antenatal and postnatal anxiety: systematic review and meta-analysis. Br J Psychiatry. 2017;210(5):315–23. 28302701 10.1192/bjp.bp.116.187179

[3] Doraiswamy S, Jithesh A, Chaabane S, Abraham A, Chaabna K, Cheema S. Perinatal mental illness in the middle East and North Africa region—A systematic overview. Int J Environ Res Public Health. 2020;17(15):5487. 32751384 10.3390/ijerph17155487 PMC7432515

[4] Bayrampour H, McDonald S, Tough S. Risk factors of transient and persistent anxiety during pregnancy. Midwifery. 2015;31(6):582–9. 25823754 10.1016/j.midw.2015.02.009

[5] Araji S, Griffin A, Dixon L, Spencer S-K, Peavie C, Wallace K. An overview of maternal anxiety during pregnancy and the post-partum period. J Mental Health Clin Psychol. 2020;4(4). https://www.mentalhealthjournal.org/articles/an-overview-of-maternal-anxiety-during-pregnancy-and-the-post-partum-period.html.

[6] Alipour Z, Lamyian M, Hajizadeh E. Anxiety and fear of childbirth as predictors of postnatal depression in nulliparous women. Women Birth. 2012;25(3):e37–43. 21959041 10.1016/j.wombi.2011.09.002

[7] Farré-Sender B, Torres A, Gelabert E, Andrés S, Roca A, Lasheras G, et al. Mother–infant bonding in the postpartum period: assessment of the impact of pre-delivery factors in a clinical sample. Arch Women Ment Health. 2018;21:287–97. 10.1007/s00737-017-0785-y 29046965

[8] Míguez MC, Fernández V, Pereira B, Depresion Postparto Y Factores Asociados, En Mujeros Con Embarazos de Riesgo. Behav Psychology/Psicologia Conductual. 2017;25(1). https://www.behavioralpsycho.com/wp-content/uploads/2018/10/03.Miguez_25-1.pdf.

[9] Ding X-X, Wu Y-L, Xu S-J, Zhu R-P, Jia X-M, Zhang S-F, et al. Maternal anxiety during pregnancy and adverse birth outcomes: a systematic review and meta-analysis of prospective cohort studies. J Affect Disord. 2014;159:103–10. 24679397 10.1016/j.jad.2014.02.027

[10] Nurrizka RH, Nurdiantami Y, Makkiyah FA. Psychological outcomes of the COVID-19 pandemic among pregnant women in indonesia: a cross-sectional study. Osong Public Health Res Perspect. 2021;12(2):80. 33979998 10.24171/j.phrp.2021.12.2.05 PMC8102875

[11] Brunton RJ, Dryer R, Saliba A, Kohlhoff J. The initial development of the Pregnancy-related anxiety scale. Women Birth. 2019;32(1):e118–30. 29859678 10.1016/j.wombi.2018.05.004

[12] Boateng GO, Neilands TB, Frongillo EA, Melgar-Quiñonez HR, Young SL. Best practices for developing and validating scales for health, social, and behavioral research: a primer. Front Public Health. 2018;6:149. 29942800 10.3389/fpubh.2018.00149 PMC6004510

[13] Hair JF, Black WC, Babin BJ, Anderson RE, Tatham RL. Multivariate data analysis (7. Baskı). Pearson Hallahan, TA, Faff RW, McKenzie MD. (2004) An Empirical Investigation of Personal Financial Risk Tolerance Financial Services Review-Greenwıch. 2010;13(1):57–78.

[14] Sousa VD, Rojjanasrirat W. Translation, adaptation and validation of instruments or scales for use in cross-cultural health care research: a clear and user‐friendly guideline. J Eval Clin Pract. 2011;17(2):268–74. 20874835 10.1111/j.1365-2753.2010.01434.x

[15] Brislin RW. Back-translation for cross-cultural research. J Cross-Cult Psychol. 1970;1(3):185–216.

[16] Polit DF, Beck CT, Owen SV. Is the CVI an acceptable indicator of content validity? Appraisal and recommendations. Res Nurs Health. 2007;30(4):459–67. 17654487 10.1002/nur.20199

[17] Lawshe CH. A quantitative approach to content validity. Pers Psychol. 1975;28(4). https://parsmodir.com/wp-content/uploads/2015/03/lawshe.pdf.

[18] Muthén L. Mplus User’s Guide. Los Angeles, CA: Muthén & Muthén; 1998.

[19] Brown TA. Confirmatory factor analysis for applied research. Guilford Publication; 2015.

[20] Zitzmann S, Helm C, Hecht M. Prior specification for more stable bayesian Estimation of multilevel latent variable models in small samples: A comparative investigation of two different approaches. Front Psychol. 2021;11:611267. 33569026 10.3389/fpsyg.2020.611267 PMC7868428

[21] Lt H, Bentler PM. Cutoff criteria for fit indexes in covariance structure analysis: conventional criteria versus new alternatives. Struct Equation Modeling: Multidisciplinary J. 1999;6(1):1–55.

[22] Kline RB. Principles and practice of structural equation modeling. Guilford Publication; 2023.

[23] Anuar A, Sadek DM. Validity test of lean healthcare using lawshe’s method. Int J Supply Chain Manage. 2018;7(6):197–203.

[24] Feng FU, Xiaotong L, Yuhang Z, Juanjuan Y, Mingyue M, X inxing D, Xinmin M. Current status and influencing factors of pregnancy related anxiety in Chinese pregnant women: a cross sectional study. 2025, PREPRINT availabel at Research Square 10.21203/rs.3.rs-5781802/v1.

[25] Xie T, Han L, Wu J, Dai J, Fan X, Liu J, et al. Psychometric evaluation of the pregnancy-related anxiety questionnaire—revised 2 for Chinese pregnant women. Midwifery. 2022;112:103411. 35779320 10.1016/j.midw.2022.103411

[26] Kurt G, Arslan H. Turkish version of the Pregnancy-related anxiety scale: A psychometric study. Perspect Psychiatr Care. 2021;57(1):157–66. 32458429 10.1111/ppc.12537

[27] Dryer R, Brunton R, He D, Lee E. Psychometric properties of the Pregnancy-Related anxiety Scale—Screener. Psychol Assess. 2022;34(5):443. 35084891 10.1037/pas0001110

[28] Bright KS, Premji SS. Cross-cultural perspectives of pregnancy-related anxiety. Pregnancy-related anxiety: Routledge; 2021. pp. 143–57.

