# Supplementary material for: Validation of the pregnancy-related anxiety scale (PrAS) for pregnant women in Indonesia
Source: BMC Pregnancy Childbirth. 2025 Sep 30;25:968. doi: 10.1186/s12884-025-08036-7 (PMC12487145; doi:10.1186/s12884-025-08036-7)
Supplement: Supplementary file 1 — Supplementary Material 1. [file 12884_2025_8036_MOESM1_ESM.pdf]

UNIVERSITAS INDONESIA
FAKULTAS ILMU KEPERAWATAN

Gedung Fakultas Ilmu Keperawatan
Kampus UI, Depok Jawa Barat 16424
T. 62 21 788 49 120 **F.** 62 21 786 41 24
E. publicrelation-nursing@ui.ac.id
nursing.ui.ac.id

SURAT KETERANGAN LOLOS KAJI ETIK
Nomor: KET-185/UN2.F12.D1.2.1/PPM.00.02/2023

Yang bertanda tangan di bawah ini
nama : Prof. Dra. Setyowati, S.Kp., M.App.Sc., Ph.D.
jabatan : Ketua Komite Etik FIK UI
unit kerja : Fakultas Ilmu Keperawatan Universitas Indonesia

menerangkan bahwa

Komite Etik Penelitian, Fakultas Ilmu Keperawatan Universitas Indonesia dalam upaya melindungi hak asasi dan kesejahteraan subjek penelitian keperawatan, telah mengkaji dengan teliti proposal berjudul

Validation of the Pregnancy Related Anxiety Scale for Pregnant Woman in Indonesia: A Descriptive, Cross-Sectional Study

nama peneliti utama : **Dr. Wiwit Kurniawati, M.Kep., Sp.Mat.**
nama institusi : **Fakultas Ilmu Keperawatan Universitas Indonesia**

Komite telah menyatakan bahwa proposal penelitian ini layak dilaksanakan sesuai dengan prinsip etik penelitian.

18 Juli 2023
Ketua Komite Etik FIK UI

Prof. Dra. Setyowati, S.Kp., M.App.Sc., Ph.D.
NIP 195404271977032001
